# Use of time in chronic obstructive pulmonary disease: Longitudinal associations with symptoms and quality of life using a compositional analysis approach

**DOI:** 10.1371/journal.pone.0214058

**Published:** 2019-03-21

**Authors:** Hayley Lewthwaite, Tim Olds, Marie T. Williams, Tanja W. Effing, Dorothea Dumuid

**Affiliations:** 1 Alliance for Research in Exercise, Nutrition and Activity, School of Health Sciences, University of South Australia, Adelaide, South Australia, Australia; 2 College of Medicine & Public Health, Flinders University, Bedford Park, South Australia, Australia; 3 Department of Respiratory Medicine, Southern Adelaide Local Health Network, Bedford Park, South Australia, Australia; Universite de Bretagne Occidentale, FRANCE

## Abstract

**Background and objectives:**

This study explored whether, for people with chronic obstructive pulmonary disease (COPD), changes to the 24-hour composition of physical activity (PA), sedentary behaviour (SB) and sleep were associated with changes in symptoms and health-related quality of life (HRQoL); and how time re-allocations between these behaviours were associated with changes in outcomes.

**Methods:**

This study pools data on people with COPD drawn from two previous studies: a randomised controlled trial of cognitive behavioural therapy and pulmonary rehabilitation and a usual care cohort. Participants recalled behaviours and completed symptom and HRQoL assessments at baseline (T0) and four months (T1). Linear mixed-effects models (pooled control/intervention samples) predicted changes in outcomes from T0 to T1 with a change to the 24-hour behaviour composition; compositional isotemporal substitution predicted change in outcomes when re-allocating time between behaviours.

**Results:**

Valid data were obtained for 95 participants (forced expiratory volume in one second %predicted = 49.6±15.3) at T0 and T1. A change in the 24-hour behaviour composition was associated with a change in anxiety (p<0.01) and mastery (p<0.01), but not breathlessness, depression or fatigue. When modelling time re-allocation with compositional isotemporal substitution, more time re-allocated to higher intensity PA or sleep was associated with favourable changes in outcomes; re-allocating time to SB or light PA was associated with unfavourable changes in outcomes. The direction of association, however, could not be determined.

**Conclusion:**

To improve the overall health and wellbeing of people with COPD, intervention approaches that optimise the composition of PA, SB *and* sleep may be beneficial.

## Introduction

People with chronic obstructive pulmonary disease (COPD) are markedly less active than their age-matched healthy peers [1, 2], increasing their risk of hospitalisation and premature death [3]. Over the last decade, optimising levels of physical activity (PA) has been a predominant focus of research. In particular, increasing higher intensity structured PA such as gym-based exercise, walking or cycling has been prioritised [4]. The recommended amounts of daily time for these higher intensity activities, however, comprise only a small portion of each day (around 2%) [5]. Despite the known health benefits in the general population [6], few studies have considered the mix of movement behaviours: light intensity PA (LPA), sedentary behaviour (SB) and sleep, that people with COPD participate in over the remainder of the day.

The importance of considering the mix of behaviours over the entire day—the *24-hour movement behaviour composition*—has recently been highlighted [7]. Compared to people with mild COPD, those with severe disease spent around four additional hours per day in SB (15 h/d per day *versus* 11 h/d) [7]. This occurred at the expense of LPA, most notably, household chores (~3.5 h/d less) [7]. Preventing shifts of LPA or sleep to SB may be an achievable goal for people with COPD to achieve meaningful health benefits. No study to date has explored whether, and by how much, changes in the time spent in 24-hour movement behaviours are associated with health outcomes among people with COPD.

Using pre-existing data specific to people with COPD, this study aimed to answer two research questions:

1. Were changes to the 24-hour movement behaviour composition associated with changes in symptoms or health-related quality of life (HRQoL); and
2. Were re-allocations of time between movement behaviours associated with changes in these outcomes.

It was hypothesised that time re-allocations towards sleep and PA would be favourably associated with symptoms and HRQoL, and re-allocations towards SB would be unfavourably associated.

## Methods

This study is a secondary data analysis, pooling data on people with COPD drawn from two studies: a pragmatic randomised controlled trial (RCT) (ACTRN12611000292976) and a separate usual care cohort [7]. The RCT compared comprehensive pulmonary rehabilitation (PR) with or without cognitive behavioural therapy (CBT) on a range of health outcomes. Participants for the usual care cohort completed identical assessment protocols and timelines to those included in the RCT. Participants for the RCT were recruited from the Repatriation General Hospital (RGH) Adelaide PR clinic from 2011 to 2014. The usual care cohort was recruited from 2014 to 2015 from the RGH respiratory clinic research database; patients had previously provided written consent to be contacted for future research. The Human Research Ethics Committee of the University of South Australia (P153/07) and Southern Adelaide Local Health Network (P56/07) granted ethical approval. The focus of this paper is not on comparing the different groups, which will be the subject of another planned analysis, but on the relationship between changes in time use and changes in health outcomes in pooled data from all participants in both studies.

Participants were included in the RCT or usual care cohort if they had at least moderate COPD confirmed by spirometry [8]. Participants were excluded if they: 1) had cognitive or memory impairments (Mini-Mental State Exam [9] <23/30); 2) were participating in a concurrent research study; 3) registered for lung reduction surgery or lung transplantation; 4) were clinically unstable; or 5) had coexisting medical conditions contraindicated with exercise. Participants for the usual care cohort were additionally excluded if they had participated in PR within two years prior to recruitment. For the current study, with identical eligibility criteria, participant data from the pragmatic RCT and usual care cohort were pooled.

### Sample size

Designing prospective studies for testing the impact of time re-allocation on health outcomes is problematic, especially in people living with chronic disease such as COPD. We identified datasets from our past studies, which included variables for use of time and a range of health outcomes for at least two occasions. Drawing on data from these two previous studies, 95 participants (RCT: PR+CBT n = 34, PR alone n = 30; usual care cohort: n = 31) were eligible for inclusion in the current study. With a sample size of 95, this study was sufficiently powered to detect a large correlation (Model 1, f^2^ = 0.28; Model 2, f^2^ = 0.33) between use of time and outcomes (while controlling for covariates) with alpha of 0.01 and power of 0.8. Power analyses were performed using G*Power version 3.0.10 (Germany).

### Measures

#### Exposure: Use of time

The Multimedia Activity Recall for Children and Adults (MARCA) was used to assess 24-hour movement behaviours, providing detail on activity type and intensity [10]. The MARCA has shown good convergent validity (*r* = 0.66–0.74) with triaxial accelerometers for assessment of PA in people with COPD [7, 11], and with the ActivPAL3 for assessment of SB in older adults (*r* = 0.49–0.67) [12]. In people with COPD, the MARCA has demonstrated good test-retest reliability for activities grouped into common activity types (ICC = >0.88) [11].

During two 30-minute computer assisted telephone interviews, participants recalled every activity that they did over the previous two days each time. Recalled activities were matched to over 300 activities available for selection within the MARCA software [10]. Anchor points were used (e.g., meal times) to segment the day and activities were recalled with a resolution of five minutes or more (i.e., within a five minute sampling frame) [10]. Activity intensity was determined by linking recalled activities to activities within compendia with energy expenditure estimates [13]. Data were captured to include at least one weekday and one weekend day, subsequently weighted to be representative of a typical week structure.

#### Outcomes

Breathlessness was assessed during an in-clinic structured interview (details described elsewhere [14]) using the Multidimensional Dyspnea Profile (MDP) single score for affective distress (A1), recommended by developers when circumstances permit a single rating score [15, 16]. Unpleasantness of breathlessness was rated on average over the previous two weeks (zero = ‘neutral’ to 10 = ‘unbearable’).

Anxiety and depressive symptoms were assessed with the Hospital Anxiety and Depression Scale (HADS) [17], self-administered by participants. The HADS subdomains have demonstrated good test-retest reliability (*r* = >0.70) and strong sensitivity and specificity (0.70–0.90) [18].

Health-related quality of life was assessed with the Chronic Respiratory Disease Questionnaire self-administered format (CRQ-SAS) [19]. Fatigue and mastery subdomains were included in analyses. The CRQ-SAS has shown good test-retest reliability (ICC 0.83–0.95) [19] and high sensitivity [20].

#### Covariates

Baseline outcome values, and participants’ COPD status (BODE index [21]), comorbid burden (COPD-specific comorbidity test [22]), smoking status, Index of Relative Socio-economic Disadvantage (IRSD), age and sex at baseline were included as covariates. The IRSD is a postcode-level umbrella measure of socio-economic status incorporating income, education and occupation. Data were obtained during in-clinic assessments at each time point (COPD status) or by retrospective medical record review.

### Statistical analysis

Participants were described by basic socio-demographic and disease characteristics at baseline. Movement behaviours recalled by participants at T0 and T1 were grouped into:

1. Energy expenditure (EE) bands **(Model 1)**; or
2. Common activity types/‘superdomains’ **(Model 2)** (Table 1).

**Table 1:** Criteria for collating activities captured by the MARCA into four energy expenditure bands by activity intensity (Model 1), or nine common ‘superdomains’ by activity type (Model 2).

**Criteria for grouping by activity intensity: Model 1**		
	**Energy expenditure bands**	**Activity intensity requirement**
**1**	MVPA	≥3 METs
**2**	LPA	1.6–2.9 METs
**3**	SB	≤1.5 METs
**4**	Sleep	Based on self-report sleep and wake times
**Criteria for grouping by activity type: Model 2**		
	**‘Superdomain’**	**Activity types categorised into ‘superdomain’**
**1**	Physical activity	Sports/exercise
		Active transport
**2**	Chores	Indoor chores
		Outdoor chores
**3**	Self-care	Grooming/bathing
		Eating
**4**	Socio-cultural	Socialising
		Communicating
		Religious
		Other cultural
**5**	Transport	Passive transport (car, public transport)
**6**	Work/study	Occupational, non-screen
**7**	Screen time	Television
		Computer use
**8**	Quiet time	Reading
		Non-reading
**9**	Sleep	

**Key**: LPA, light intensity physical activity; MET, metabolic equivalent of task where 1 MET is equivalent to around 3.5 ml of oxygen consumed per kg of body mass per minute; MVPA, moderate to vigorous intensity physical activity; SB, sedentary behaviour

Dependent sample t-tests assessed change over time (T0 to T1) for time committed to different movement behaviours (Model 1 and Model 2) and symptom/HRQoL outcomes. Alpha was set at 0.05. Analyses were conducted using Statistical Package for the Social Sciences (SPSS) version 22.

The time participants spent in different movement behaviours (e.g., MVPA, LPA, SB and sleep in Model 1) was expressed as proportions of the complete 24-hour day—*the 24-hour movement behaviour composition*. The mean time spent in each movement behaviour at T0 and T1 was first expressed as the geometric mean. The geometric means were linearly adjusted to sum to 1, or 100%, expressing the parts as proportions. The values were rescaled to sum to 1440 minutes [23].

The 24-hour movement behaviour composition has specific statistical properties, which renders it unsuitable for inclusion in most multivariate statistical models. The sum of all parts must equal 24 hours; if one part is increased, other parts must decrease to compensate. This means the compositional parts are co-dependent and perfectly multi-collinear. Compositional data analysis (CoDA) overcomes this by expressing the composition as a set of log ratios. A particularly useful log-ratio transformation is the isometric log-ratio transformation (*ilr*) [23, 24]. The presence of zero values in any compositional parts prevents the applications of log ratios, as the logarithm of zero is undefined. Before expressing the movement behaviour composition as *ilrs*, zeros were replaced by 65% of the 5-minute MARCA sampling frame (i.e., 3.25 min) [25].

To answer the first research question *‘Was a change in the 24-hour movement behaviour composition associated with a change in outcomes*?*’*, linear mixed-effects models were used. The change in the 24-hour movement behaviour composition (as a set of *ilrs*) from T0 to T1 was the independent variable and the change in each outcome the dependent variable. Models were adjusted for covariates. Regression models were performed for Model 1, where movement behaviours were defined by EE bands, and for Model 2, where movement behaviours were defined by activity ‘superdomains’. To account for nesting within groups random intercepts were used. Analyses were conducted in the statistical package software R (R Core Team, Vienna) using the statistical packages lme4, Compositions and zCompositions. Sequential Bonferroni corrections were applied to alpha [26].

To answer the second research question *‘Were re-allocations between behaviours associated with a change in outcomes*?*’*, compositional isotemporal substitution analysis was used [27]. Compositional isotemporal substitution analysis used the regression models from above (Model 1 and Model 2) to predict the change in outcomes when a fixed duration of time was re-allocated between the domains of the composition (e.g., adding 30 minutes time to the time spent in MVPA) by:

1. Drawing the equivalent amount of time from all remaining movement behaviour domains of the composition in their relative proportions: **‘one-for-remaining’ re-allocation**. That is, in Model 1, if we re-allocated 30 minutes of time to MVPA, we drew that time pro rata from each of the remaining EE bands in the composition (e.g., if sleep comprised 33% of the remainder of the day, one third of the 30 minutes, 10 minutes, was drawn from sleep; if LPA comprised 20%, then 20% of the 30 minutes, 6 minutes, was drawn from LPA, and so on); or
2. Drawing the equivalent amount of time from one other movement behaviour domain: ‘**one-for-one’ re-allocation.** That is, using Model 1 as the example again, if we re-allocated 30 minutes to MVPA, then we took this time directly from SB *or* from LPA *or* from sleep.

## Results

**Fig 1:**
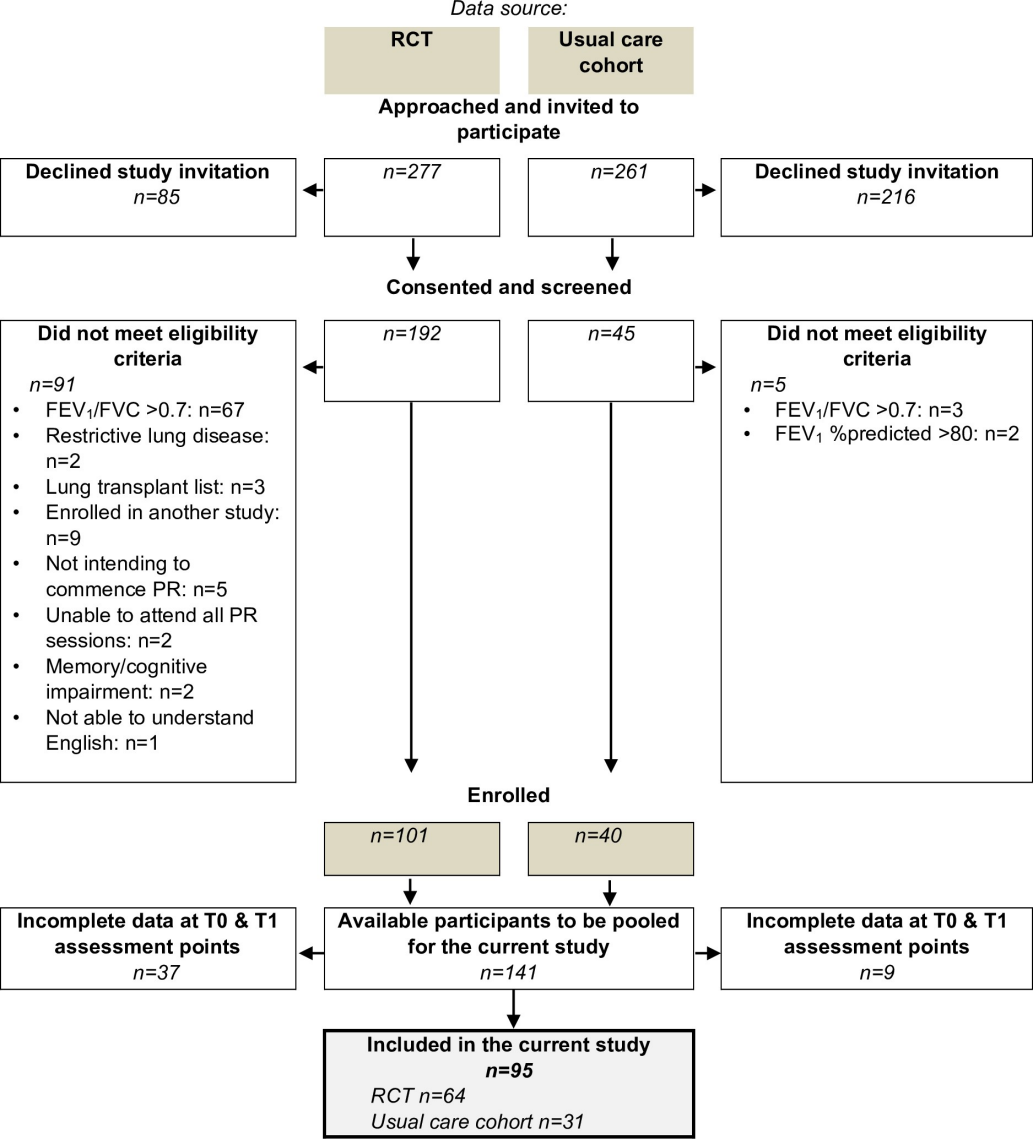
Participant flow. FEV_1_, forced expiratory volume in one second; FVC, forced vital capacity; RCT, randomised control trial.

Of the 141 participants included in the RCT (n = 101) or usual care cohort (n = 40), 95 (67%) were included in the current study (Fig 1). Participants were predominantly male (63%), 70.5±6.8 years of age with severe COPD (FEV_1_%predicted = 49.6±15.3) [8]) (Table 2). There was no significant change in participants’ use of time, with the exception of committing more time to work/study at T1 (T0: 46±65 min/d; T1: 70±82 min/d, p = 0.01). There were favourable changes in participants’ anxiety (p = 0.01) and depression (p = 0.03) (Table 3).

**Table 2:** Participant characteristics at baseline.

		Data source:		
Participant characteristic	Pooled sample (n = 95)	PR+CBT (n = 34)	PR alone (n = 30)	UC cohort (n = 31)
Age (years)	70.5 (±6.8)	71 (±5.5)	70.1 (±8.5)	70.3 (±6.5)
Sex (%male)	63%	53%	70%	77%
IRSD	989 (±70.5)	982 (±64.6)	1001 (±75.1)	986 (±73)
BMI (kg/m^2^)	28.1 (±5.4)	27.2 (±5.9)	27.8 (±5.7)	29.8 (±4.3)
FEV_1_%predicted	49.6 (±15.3)	46.5 (±12.3)	47.2 (±16.6)	55.3 (±15.8)
FEV_1_/FVC	0.44 (±0.13)	40.5 (±11.4)	41.5 (±14.3)	49 (±12.3)
mMRC (n = , %)				
0	8 (8.4%)	0 (0%)	3 (10%)	5 (16.1%)
1	38 (40%)	19 (55.9%)	10 (33.3%)	9 (29%)
2	21 (22.1%)	9 (26.5%)	5 (16.7%)	7 (22.6%)
3	16 (16.8%)	6 (17.6%)	6 (20%)	4 (12.9)%
4	12 (12.6%)	0 (0%)	6 (20%)	6 (19.4%)
6MWD (m) *(n = 94)*	385.2 (±117.7)	396.1 (±98.1)	386.5 (±133.4)	372.4 (±123.3)
BODE (n = , %)				
0	10 (10.5%)	1 (2.9%)	4 (13.3%)	5 (16.1%)
1	19 (20%)	9 (26.5%)	4 (13.3%)	6 (19.4%)
2	20 (21.1%)	8 (23.5%)	6 (20%)	6 (19.4%)
3	11 (11.6%)	4 (11.8%)	4 (13.3)	3 (9.7%)
4	13 (13.7%)	7 (20.6%)	3 (10%)	3 (9.7%)
5	7 (7.4%)	1 (2.9%)	3 (10%)	3 (9.7%)
6	9 (9.5%)	2 (5.9%)	4 (13.3%)	3 (9.7%)
7	3 (3.2%)	2 (5.9%)	1 (3.3%)	0 (0%)
8	3 (3.2%)	0 (0%)	1 (3.3%)	2 (6.5%)
9	0 (0%)	0 (0%)	0 (0%)	0 (0%)
10	0 (0%)	0 (0%)	0 (0%)	0 (0%)
COTE Index (Median, IQR)	1.00 (3)	1 (2%)	1 (3%)	1 (3%)
Smoking status (n = , %current)	17 (18%)	7 (20.6%)	3 (10%)	7 (22.6%)
MMSE	29.2 (±1.8)	29.5 (±1.1)	29.2 (±1.7)	28.8 (±2.3)

**Key:** 6MWD, six-minute walk distance; BMI, body mass index; BODE, composite score of BMI, airflow obstruction, dyspnoea and exercise capacity; CBT, cognitive behavioural therapy; COTE, COPD-specific comorbidity test; FVC, forced vital capacity; FEV_1_, forced expiratory volume in one second; IRSD, Index of Relative Socio-economic Disadvantage; mMRC, modified Medical Research Council dyspnoea score; MMSE, Mini Mental State Exam; PR, pulmonary rehabilitation; UC, usual care. Results expressed as mean (±SD) unless otherwise reported

**Table 3:** Time spent in movement behaviours, symptoms and health-related quality of life at T0 and T1 assessment points.

	T0 *Pooled sample*: *n = 95*	T1 *Pooled sample*: *n = 95*	*test for sig*, *p =*
**Behaviour domains**			
**Energy expenditure bands** (min/d): Model 1			
MVPA	93 (71)	91 (78)	0.74
LPA	272 (96)	276 (110)	0.68
SB	597 (124)	591 (148)	0.61
Sleep	477 (70)	482 (74)	0.48
**‘Superdomains’** (min/d): Model 2			
Sleep	477 (70)	482 (74)	0.48
Physical activity	26 (31)	27 (27)	0.65
Chores	188 (93)	173 (91)	0.08
Quiet time	155 (85)	147 (89)	0.37
Screen time	261 (122)	246 (118)	0.21
Self-care	139 (27)	137 (32)	0.63
Socio-cultural	105 (66)	104 (66)	0.91
Transport (passive)	43 (33)	54 (61)	0.06
Work/study*	46 (65)	70 (82)	0.01
**Outcome**			
Breathlessness	4.58 (2.38)	4.38 (2.28)	0.43
Anxiety*	6.61 (4.04)	5.82 (4.1)	0.01
Depression*	5.76 (3.68)	5.05 (3.43)	0.03
Fatigue	3.93 (1.29)	4.13 (1.4)	0.07
Mastery	4.97 (1.28)	5.18 (1.39)	0.09

**Key:** EE, energy expenditure; LPA, light intensity physical activity; MVPA, moderate to vigorous intensity physical activity; SB, sedentary behaviour

Results expressed as arithmetic mean (±SD). Significance level p<0.05.

*significant change over time

The first aim of this study was to explore whether a change in the 24-hour composition of movement behaviours from T0 to T1 was associated with a change in outcomes of breathlessness unpleasantness, fatigue, anxiety, depression or mastery. In both **Model 1** (movement behaviours grouped by EE bands) and **Model 2** (movement behaviours grouped by activity ‘superdomains’), changes in the 24-hour movement behaviour composition from T0 to T1 were associated with a change in anxiety (Model 1, p = 0.007; Model 2, p = 0.007) and mastery (Model 1, p<0.001, Model 2, p = 0.002), but not breathlessness (Model 1, p = 0.2; Model 2, p = 0.4), depression (Model 1, p = 0.13; Model 2, p = 0.4) or fatigue (Model 1, p = 0.2; Model 2 p = 0.3).

The second aim of this study was to explore whether re-allocations between movement behaviours (using both **Model 1**: EE bands and **Model 2**: activity ‘superdomains’) were associated with changes in outcomes. First, we looked at what would happen if we re-allocated time to each of the EE bands **(Model 1)** by taking this time pro rata from the remaining EE bands (i.e., **‘one-for-remaining’ re-allocations**). The change in outcomes when re-allocating 30 minutes to each EE band, taking this time from the remaining EE bands of the composition in their relative proportions (i.e., **‘one-for-remaining’ re-allocations**) are presented in Table 4. Favourable changes in outcomes were observed when re-allocating time to MVPA (standardised effect size for 30 minute re-allocations: anxiety = 0.04; depression = 0.08; fatigue = 0.05; mastery = 0.07) or sleep (standardised effect size for 30 minute re-allocations: breathlessness = 0.08; anxiety = 0.06; fatigue = 0.03; mastery = 0.09). Detrimental associations were observed when time was re-allocated to SB (standardised effect size for 30 minute re-allocations: breathlessness = -0.04; anxiety = -0.05; depression = -0.01; mastery = -0.06) or LPA (standardised effect size for 30 minute re-allocations: breathlessness = -0.05; anxiety = -0.02; depression = -0.01; fatigue = -00.05; mastery = -0.06). The standardized effect sizes for all re-allocations and outcomes are available in S1 Dataset.

**Table 4:** Association with change in outcomes when 30 minutes of time was re-allocated to each energy expenditure band (when drawing time pro rata from the remainder of the energy expenditure bands in the composition).

Energy Expenditure Band	Outcome				
	Breathlessness	Anxiety	Depression	Fatigue	Mastery
↑MVPA	0.00	+0.04	+0.08	+0.05	+0.07
↑LPA	-0.05	-0.02	-0.01	-0.05	-0.06
↑SB	-0.04	-0.05	-0.01	0.00	-0.06
↑Sleep	+0.08	+0.06	0.00	+0.03	+0.09

**Key:** EE, energy expenditure; LPA, light intensity physical activity; MVPA, moderate to vigorous intensity physical activity; SB, sedentary behaviour

Results are standardised effect size when 30 minutes were re-allocated to each EE band, by taking the time pro rata from the remaining EE bands of the composition. For example, in the first row, when 30 minutes were re-allocated to MVPA by taking the time from LPA, SB and sleep in their relative proportions, there was a favourable change in depression by 0.08 standard deviations.

The direction of association has been presented so that positive effect sizes indicate favourable changes and negative effect sizes indicate unfavourable changes.

**Fig 2:**
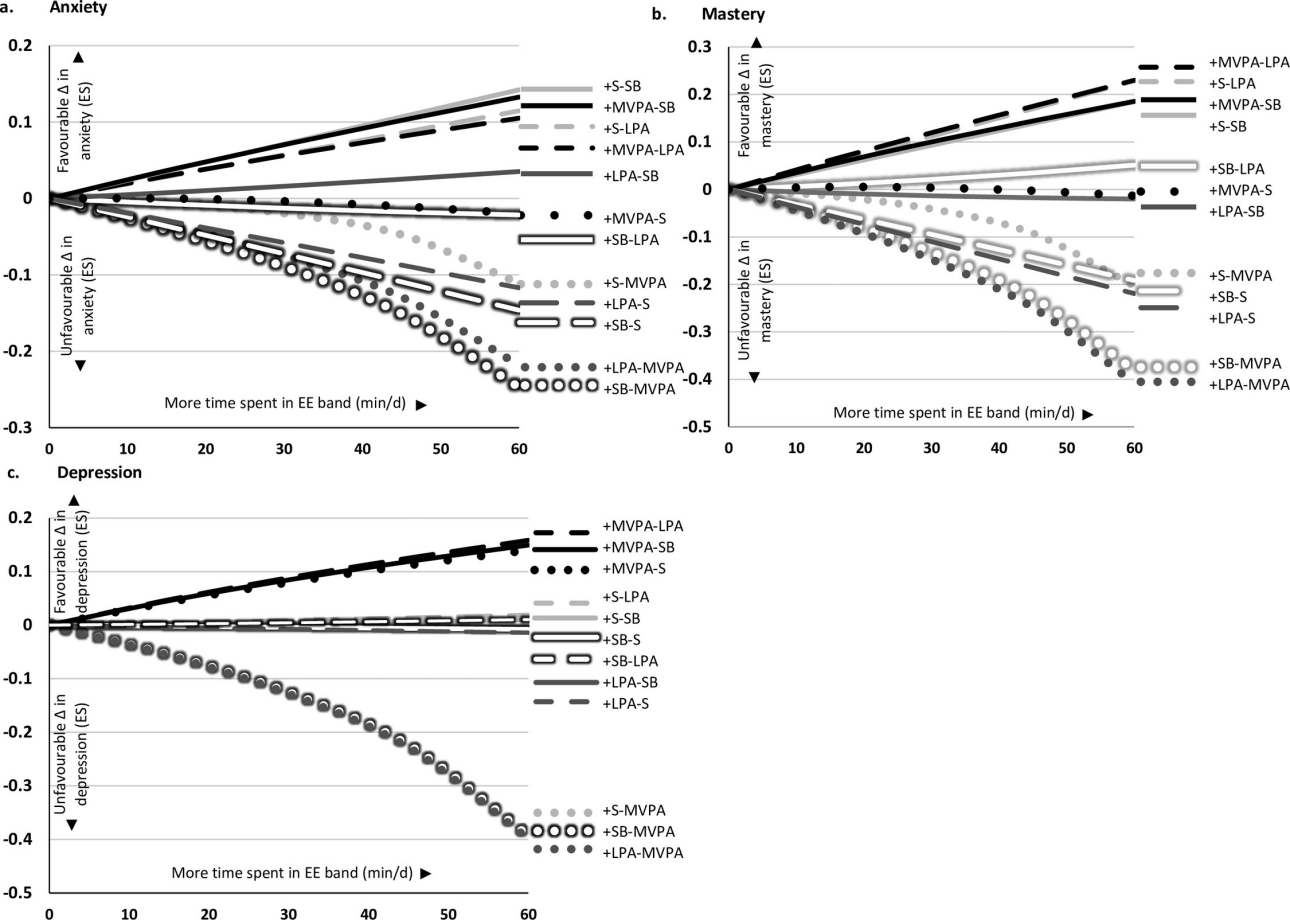
**Association with change in a) anxiety; b) mastery; and c) depression when re-allocating the time from one energy expenditure band (+) by taking the time from another energy expenditure band (-).** EE, energy expenditure; ES, effect size; LPA, light physical activity; MVPA, moderate to vigorous physical activity; S, sleep; SB, sedentary behaviour. Change (Δ) in outcome is presented as standardised effect size.

**Fig 3:**
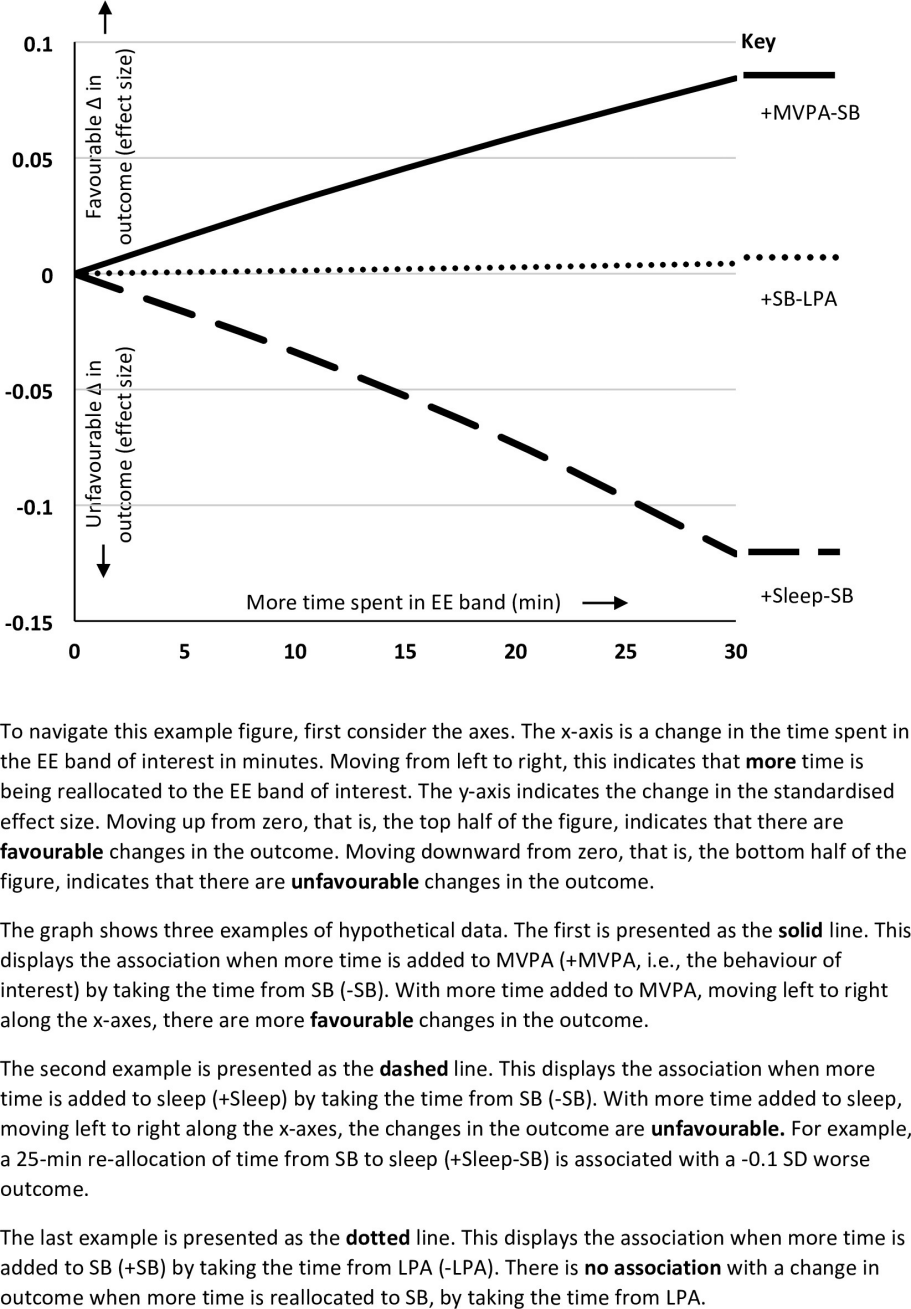
Example of re-allocation plots. EE, energy expenditure; ES, effect size; LPA, light physical activity; MVPA, moderate to vigorous physical activity; S, sleep; SB, sedentary behaviour.

Next, we looked at what would happen if we re-allocated time to each of the EE bands, by taking all of the time from one other EE band (i.e., **‘one-for-one’ re-allocations**). For example, if we added 60 minutes of time to MVPA by taking 60 minutes time from SB. The associations with anxiety, mastery, and depression when re-allocating time to each of the EE bands in the **‘one-for-one’ re-allocations** are presented in Fig 2. To navigate the plots, an example is provided in Fig 3. One-for-one re-allocations for breathlessness and fatigue are presented in S1 Fig. Favourable changes in **anxiety and mastery** were observed (Fig 2A and 2B) when MVPA or sleep replaced SB or LPA. Favourable changes in **depression** (Fig 2C) were observed when time was re-allocated to MVPA by replacing LPA, SB *or* sleep. These associations, however, did not reach significance (Model 1, p = 0.13; Model 2, p = 0.40). The standardized effect sizes for all re-allocations and outcomes are available in S1 Dataset.

**Fig 4:**
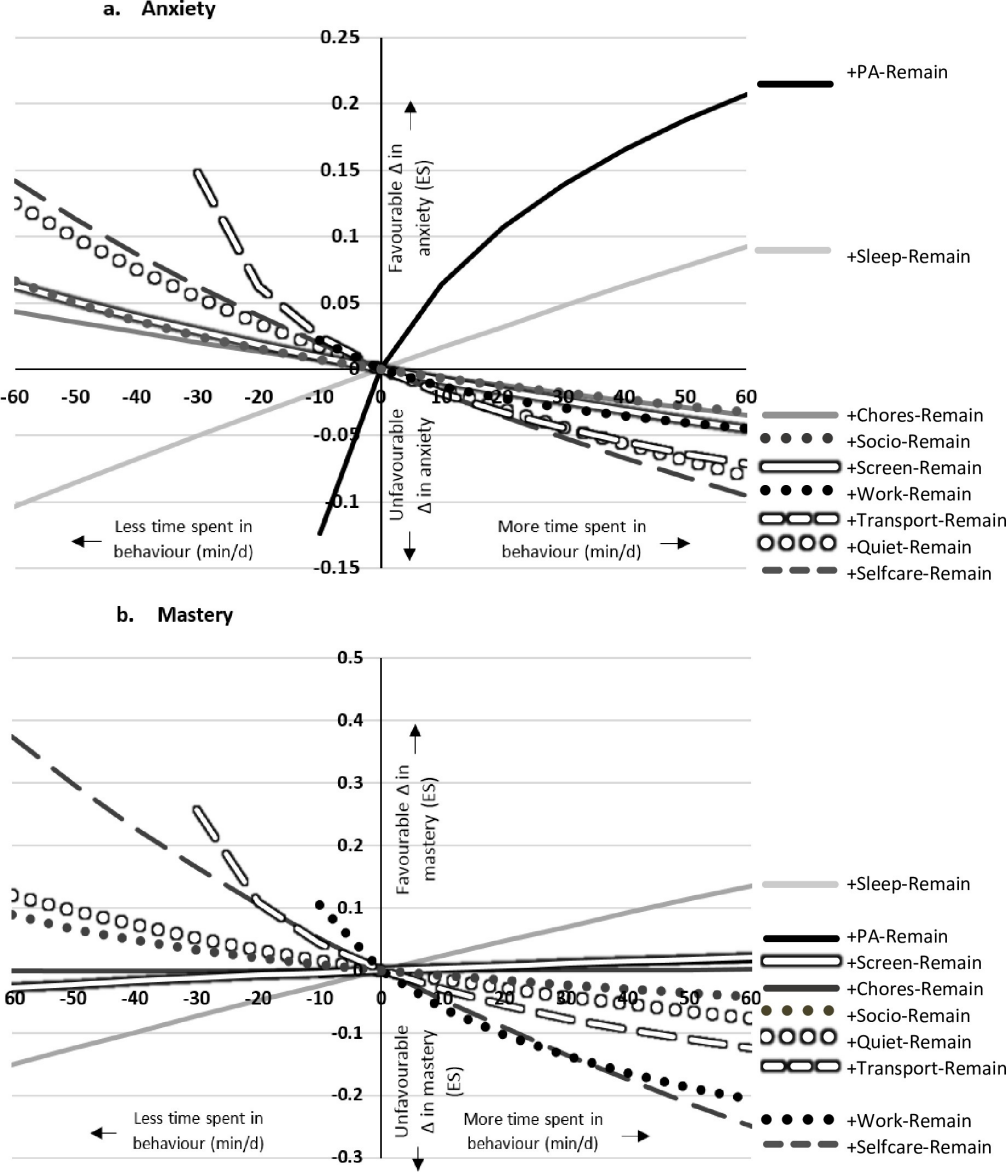
**Effect on a) anxiety; and b) mastery when re-allocating time to each MARCA activity ‘superdomain’, by taking the time from the remainder of the composition**. ES, effect size; PA, physical activity; Remain, remainder of the composition. Change (Δ) in outcome is presented as standardised effect size.

Looking at 24-hour movement behaviours grouped by common activity types in MARCA ‘superdomains’ **(Model 2)** provides greater detail as to what specific behaviours were associated with positive or negative changes to outcomes. Similar to Model 1, favourable changes in outcomes were observed when re-allocating time to PA or sleep ‘superdomains’ by taking this time from the remaining ‘superdomains’ of the composition (i.e., **‘one-for-remaining’ re-allocations**). There were a number of more interesting observations. When time was re-allocated to: 1) self-care, this was associated with unfavourable changes in all outcomes; 2) passive transport or work/study, this was associated with unfavourable changes in breathlessness and mastery. Association with outcomes of anxiety and mastery are presented in Fig 4. Breathlessness unpleasantness, depression and fatigue are presented in S2 Fig. The standardized effect sizes for all re-allocations and outcomes are available in S1 Dataset.

## Discussion

For this sample of participants with at least moderate COPD, a change in the 24-hour movement behaviour composition was associated with a change in anxiety and mastery. When re-allocating time between movement behaviours we identified a number of interesting patterns:

1. Re-allocating time to higher intensity PA or sleep was associated with favourable changes in symptoms and HRQoL;
2. Re-allocating time to LPA or SB was associated with unfavourable changes in symptoms and HRQoL; and
3. Re-allocating time to specific activity types of self-care, passive transport and work/study was unfavourably associated with symptoms and HRQoL.

### Comparison with other studies

The custom of research has been to look at movement behaviours in isolation, in particular MVPA, and their relationship with health [23, 28]. This approach fails to account for the co-dependency of behaviours [28]. To increase the time committed to one part of the composition (e.g., MVPA), time must be drawn from one or more other parts (LPA, SB and/or sleep), which may have concomitant health impacts [23, 28]. Recently, studies that look at the impact of time-reallocations on health outcomes using isotemporal substitution (such as compositional isotemporal substitution or the method proposed by Mekary et al. [29]) have been emerging, which aim to account for this confounding [6].

A systematic scoping review of isotemporal substation studies undertaken by members of the research team identified 56 studies, of which, six were in adult clinical populations using the Mekary et al. isotemporal substitution method [6]. In these clinical populations (cancer n = 3; type two diabetes n = 3), re-allocating time to more active behaviours (LPA or MVPA) was associated with favourable HRQoL; few other health outcomes were explored [6]. In adults without chronic disease, there was some evidence that re-allocating sedentary time to higher intensity physical activity was associated with improved depressive symptoms [6]. The majority of studies in this field have focused on how use of time was associated with mortality or cardiometabolic risk, with time re-allocations from sedentary to more active behaviours (LPA or MVPA) associated with more favourable outcomes [6]. Few studies have explored time re-allocations with sleep [6].

The findings of this recent review are in line with those of the current study. Favourable associations were observed for depression when MVPA replaced any other behaviour (sleep, LPA or MVPA), and for anxiety and mastery, when MVPA replaced LPA or SB. For people with COPD, while not analysed with isotemporal substitution methods, the volume *and* intensity of PA have previously demonstrated positive associations with HRQoL [30, 31]. Esteban et al. (2010) reported people with COPD who committed at least four hours per week to leisure-based walking had more favourable HRQoL and mastery [30], while Jen et al. (2012) reported walking intensity to be positively associated with HRQoL [31]. The size and causal direction of these associations, however, remains unclear [3]; it may be that people with COPD who have better HRQoL are more likely to remain active.

An important finding of this study was the favourable change in health outcomes when time was re-allocated to sleep. As with PA, sleep is an important health behaviour, which has bidirectional relationships with physical and psychosocial health and wellbeing [32]. For people with COPD, a small number of studies have shown poor sleep quality/quantity to be unfavourably associated with risk of COPD exacerbation and HRQoL [33]. While again the direction of these associations cannot be ascertained, the current study and those published previously highlight the need to prioritise sleep in the management of COPD. The body of work around strategies to improve sleep in people with COPD has overwhelmingly focused on improving underlying disease mechanisms (e.g., hypoventilation, hypoxemia) [4]. There are likely a host of factors that contribute to impaired sleep in this population beyond the underlying pathophysiology, of which symptom burden and impaired HRQoL may play a role.

The unfavourable change in outcomes observed when more time was re-allocated to LPA was unexpected. Light intensity activities such as self-care may be one factor driving this. An increase in the time taken to complete self-care activities may be indicative of an increase in disease burden. On average, our group of COPD participants spent around 2.3 hours per day on self-care—slightly more than what has been observed in age-matched retired adults [34]. People with COPD have previously reported breathlessness, fatigue and cough affect their ability to wash, dress [35], prepare food and eat [36]. To engage in these activities, they intersperse with periods of rest or medication use [35, 36]. Not surprisingly, in older adults, a loss of ability to complete these activities has shown deleterious associations with important health outcomes [37].

It must also be considered that unfavourable changes in health outcomes observed when re-allocating time to LPA may be a function of the re-allocation models. When re-allocating time to light activities such as self-care, this time was drawn from all other ‘superdomains’ (PA, Sleep, Sociocultural, Chores, Screen Time, Work/Study, Transport and Quiet Time). It is possible that the unfavourable changes in health outcomes observed were not associated with the increase in these light activities *per se*, but rather with the decrease in health promoting behaviours (such as PA and sleep). This notion can be further demonstrated by looking at the EE bands. Re-allocating time to LPA by taking all of this time from sleep or MVPA was associated with unfavourable changes in health outcomes; re-allocating time to LPA by taking all of this time from SB was associated with no or favourable changes. This highlights a hierarchy to movement behaviours; some benefit is achieved when more time is spent in LPA rather than SB, but more time spent in higher intensity PA and sleep is best.

### Strengths and limitations

The strength of this study was the use of the statistical analysis approach—compositional data analysis, or CoDA [23]—to explore associations between changes in health outcomes and 24-hour time use in COPD. While a number of studies have used the isotemporal substitution method proposed by Mekary et al [29] to explore associations between use of time and health outcomes, few have used the CoDA approach, and to the authors knowledge, this is the first study to take such an approach in the COPD field. The CoDA approach permits the mix of PA, SB *and* sleep over the entire day to be included in traditional analyses [23, 28]. Studies of isotemporal substitution often do not account for time spent in sleep, despite comprising a large proportion of the day and having significant implications for health outcomes [6]. This study was further strengthened by use of the MARCA, capturing information on different activity types and their intensity. While providing detailed use of time data, the MARCA is limited by the reliance on participant recall and may over- or underestimate the time committed to some behaviours. However, the MARCA is a very reliable measure of use of time in people with COPD, and has good validity when compared to multi-sensor devices [7, 11].

This study was limited by the generalisability of the sample. The sample size was modest, and participants were exposed to different interventions (PR+CBT n = 34; PR n = 30; usual care cohort n = 31). Exposure to PR and/or CBT may have influenced the natural course of change in symptoms, HRQoL and/or behaviours. The purpose of this study was, however, to model how a change in 24-hour movement behaviours were associated with a change in health outcomes. The inclusion of participants exposed to different interventions was deliberate to add additional variance to data on which to base compositional isotemporal substitution models. There are many factors that potentially influence participants’ movement behaviours and/or health-related outcomes, including participation in interventions (such as PR), changes to medical management, ageing, disease course, weather, etc.—all of which are normal and expected exposures for people with COPD. Finally, this study explored the association between a *change* in use of time and a *change* in health outcomes. For this, we required participant data on use of time and health outcomes collected at the same two time points (T0 and four months later at T1). The direction of association, therefore, could not be reliably determined and remains to be further explored.

## Conclusion

For this group of participants with COPD, a change in the 24-hour movement behaviour composition was associated with a change in symptoms and HRQoL. Re-allocating time to higher intensity PA or sleep was associated with favourable changes. Deleterious changes were observed when more time was re-allocated to SB or light activities, such as self-care. While reducing prolonged SB is an accepted avenue towards improving health for people with COPD, with studies underway in this area, improving sleep quality/quantity remains relatively unexplored for people with COPD without a co-existing sleep disorder. Similarly, few studies have explored how to reduce the burden of everyday activities such as self-care. Targeting non-MVPA behaviours may provide a novel intervention avenue for improving the psychological, and potentially physical, health and wellbeing of people with COPD; especially relevant for people with COPD who have exercise limitations.

## Supporting information

S1 Dataset Participant raw data and standardized effect sizes for associations between time re-allocations and health outcomes. (XLSX)

S1 Fig **Association with change in a) fatigue; and b) breathlessness unpleasantness when re-allocating the time from one energy expenditure band (+) by taking the time from another energy expenditure band (-).** EE, energy expenditure; ES, effect size; LPA, light physical activity; MVPA, moderate to vigorous physical activity; S, sleep; SB, sedentary behaviour. Change (Δ) in outcome is presented as standardised effect size.
(TIFF)

S2 Fig ‘**One-for-remaining’ models: effect on a) breathlessness unpleasantness; b) depression; and c) fatigue when re-allocating time to each MARCA activity ‘superdomain’, by taking the time from the remainder of the composition.** EE, energy expenditure; ES, effect size; LPA, light physical activity; MVPA, moderate to vigorous physical activity; S, sleep; SB, sedentary behaviour. Change (Δ) in outcome is presented as standardised effect size.
(TIFF)
