# Supplementary figures and images for: Use of time in chronic obstructive pulmonary disease: Longitudinal associations with symptoms and quality of life using a compositional analysis approach
Source: PLoS One. 2019 Mar 21;14(3):e0214058. doi: 10.1371/journal.pone.0214058 (PMC6428329; doi:10.1371/journal.pone.0214058)

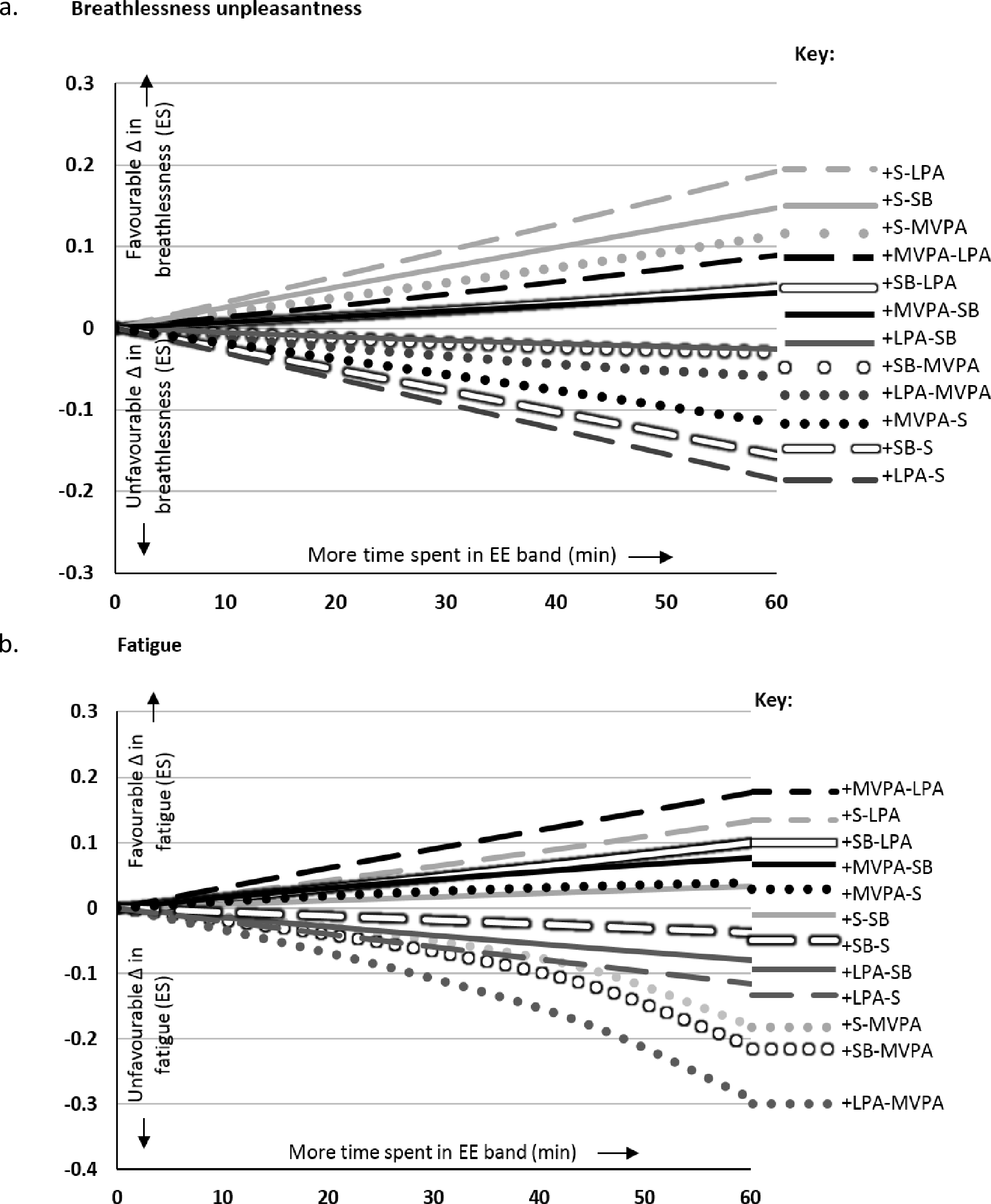

Supplement: S1 Fig — Association with change in a) fatigue; and b) breathlessness unpleasantness when re-allocating the time from one energy expenditure band (+) by taking the time from another energy expenditure band (-). EE, energy expenditure; ES, effect size; LPA, light physical activity; MVPA, moderate to vigorous physical activity; S, sleep; SB, sedentary behaviour. Change (Δ) in outcome is presented as standardised effect size. (TIFF) [file pone.0214058.s002.tiff]

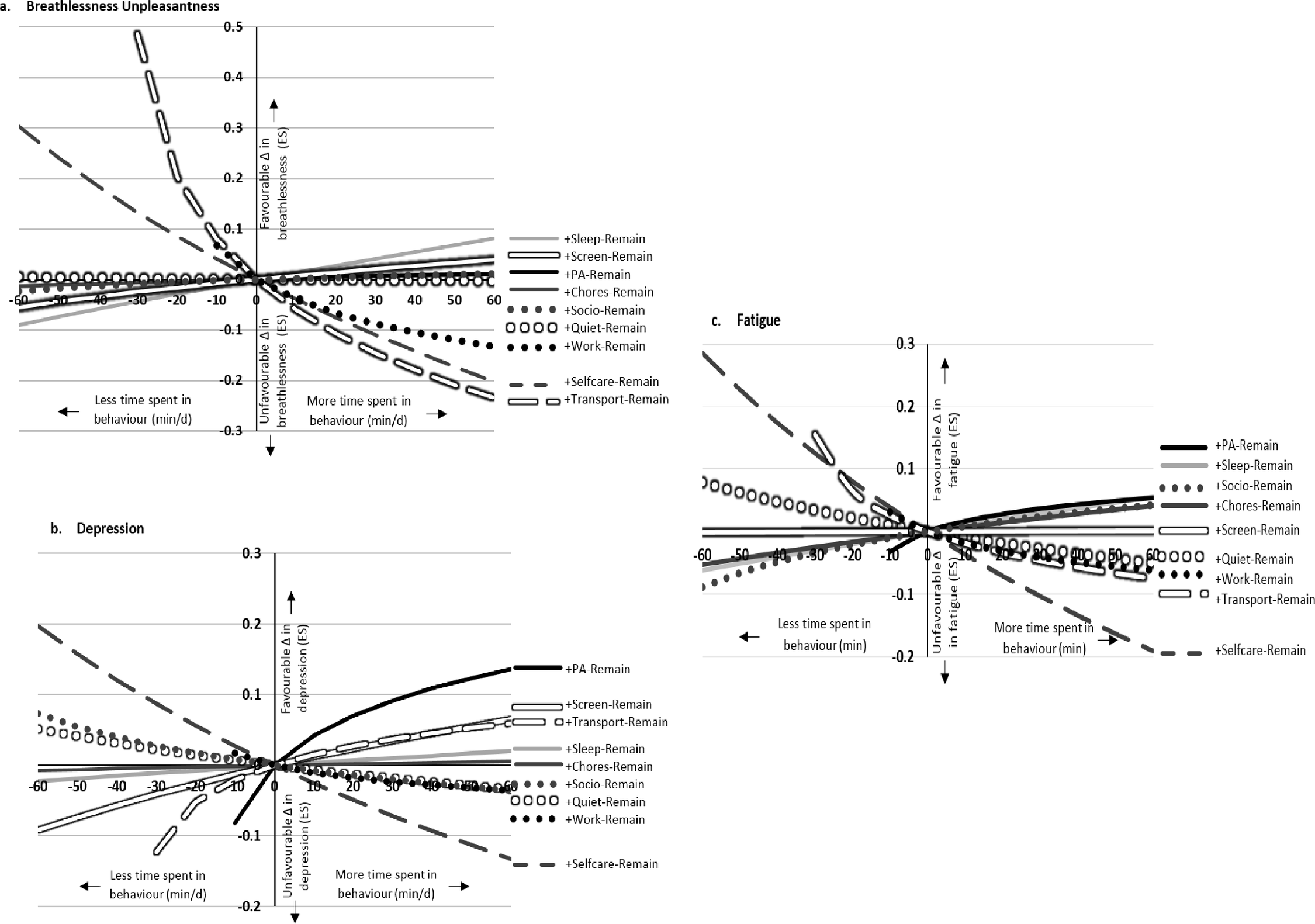

Supplement: S2 Fig — ‘One-for-remaining’ models: effect on a) breathlessness unpleasantness; b) depression; and c) fatigue when re-allocating time to each MARCA activity ‘superdomain’, by taking the time from the remainder of the composition. EE, energy expenditure; ES, effect size; LPA, light physical activity; MVPA, moderate to vigorous physical activity; S, sleep; SB, sedentary behaviour. Change (Δ) in outcome is presented as standardised effect size. (TIFF) [file pone.0214058.s003.tiff]
